# PReS13-SPK-1408: New EU scientific guidelines for JIA, SLE and GIOP from the European Medicines Agency

**DOI:** 10.1186/1546-0096-11-S2-I16

**Published:** 2013-12-05

**Authors:** R Vesely

**Affiliations:** 1 Paediatric Medicines, European Medicines Agency, London, UK

## Background: Development of EU guidelines

Guideline (GL) in the pharmaceutical legislative framework represents a harmonised EU approach based on the most up-to-date scientific knowledge to facilitate planning the overall pharmaceutical product development, the preparation of applications for marketing authorisations by the pharmaceutical industry and the assessment, approval and control of medicinal products in the European Union. It has no legal force ("soft law" non-legally binding but quasi-binding character). Alternative approaches may be taken, provided that these are appropriately justified.

## Methods - EU guideline development steps

- Development, adoption and release for consultation of concept paper.

- Preparation and release for consultation of draft guideline.

- Preparation and adoption of final guideline for publication and its implementation.

- All steps are transparent, published on EMA website http://www.ema.europa.eu/ema/.

## Results - Development of scientific guidelines for JIA, SLE and GIOP

1. Juvenile idiopathic arthritis (JIA)

EMA paediatric rheumatology expert meetings 2009, 2010, identified a need for new guideline and discussed its principles. Concept paper on the need for revision of the guideline has been released for public consultation in 2012. Draft GL is prepared to be published at the time of abstract submission.

Main principles:

- Link to adult development.

- Lowering the minimum age from 6 to 1 year, need of PK studies.

- Study design, primary and secondary endpoints, extrapolation.

- Development in JIA uveitis, vaccination sub-studie.s

- Need for pharmacovigilance studies.

2. Systemic lupus erythematosus (SLE)

Consultation with experts from PRINTO and SLE WG of PRES for the section on paediatric SLE was conducted. Draft GL on clinical investigation of medicinal products for the treatment of SLE, cutaneous lupus and lupus nephritis was released for public consultation (May 2013).

Main principles (for paediatric SLE)

- Use of PRINTO criteria in children.

- PK studies in 5 to 12 years old.

- Extrapolation from adult studies - need for CNS and renal involvement to be included.

3. Glucocorticoid induced osteoporosis (GIOP)

Existing GL for primary osteoporosis needs to be amended to add GIOP. Concept paper on the need for revision was published in 2012. Public consultation closed, draft guideline to be published.

Main principles:

- Need for treatment in GIOP, differences between paediatric and adult GIOP.

- Primary and secondary endpoints (fractures, bone mineral density, biomarkers).

- Study design (placebo control).

## Disclosure of interest

None declared.

